# Transactivation of PDGFRβ by dopamine D4 receptor does not require PDGFRβ dimerization

**DOI:** 10.1186/1756-6606-3-22

**Published:** 2010-07-26

**Authors:** Sum Shing Chi, Sandra M Vetiska, Robin S Gill, Marilyn S Hsiung, Fang Liu, Hubert HM Van Tol

**Affiliations:** 1 Department of Neuroscience, Centre for Addiction and Mental Health, University of Toronto, Toronto, M5T 1R8, Canada; 2 Department of Pharmacology, University of Toronto, Toronto, M5T 1R8, Canada; 3 Department of Psychiatry, University of Toronto, Toronto, M5T 1R8, Canada

## Abstract

Growth factor-induced receptor dimerization and cross-phosphorylation are hallmarks of signal transduction via receptor tyrosine kinases (RTKs). G protein-coupled receptors (GPCRs) can activate RTKs through a process known as transactivation. The prototypical model of RTK transactivation involves ligand-mediated RTK dimerization and cross-phosphorylation. Here, we show that the platelet-derived growth factor receptor β (PDGFRβ) transactivation by the dopamine receptor D4 (DRD4) is not dependent on ligands for PDGFRβ. Furthermore, when PDGFRβ dimerization is inhibited and receptor phosphorylation is suppressed to near basal levels, the receptor maintains its ability to be transactivated and is still effective in signaling to ERK1/2. Hence, the DRD4-PDGFRβ-ERK1/2 pathway can occur independently of a PDGF-like ligand, PDGFRβ cross-phosphorylation and dimerization, which is distinct from other known forms of transactivation of RTKs by GPCRs.

## Introduction

Receptor tyrosine kinases (RTKs) consist of a large family of receptors whose members serve a wide range of physiological functions including growth, differentiation and synaptic modulation. The members of this receptor family generally feature an extracellular ligand-binding domain, linked by a transmembrane domain to an intracellular tyrosine kinase domain, as well as several SH2 domain-binding sites. It is generally believed that the mechanism of RTK signaling involves ligand-induced dimerization of the RTK followed by cross-phosphorylation of the tyrosine-containing motifs, which subsequently interact with SH2 domain-containing molecules such as the PI3-kinase, PLC-γ, Src, SHP-2, Grb-2 and RasGAP, to effect downstream responses [1].

The large family of G protein-coupled receptors (GPCRs) activates heterotrimeric G proteins and can mediate several cellular processes, including proliferation, differentiation and survival. The ERK1/2 signaling pathway is among the major effector pathways through which GPCRs mediate their responses [2,3]. Many GPCRs engage in ERK1/2 signaling via the activation of RTKs, in a process known as transactivation [2-4]. GPCRs such as the dopamine receptors D4 (DRD4) and D2 (DRD2) [5-7], β_2 _adrenergic receptor [8], M_1 _muscarinic receptor [9], angiotensin II receptor [10], lysophosphatidic acid (LPA) receptor [11], ET1 receptor [12] and thrombin receptor [12] have been shown to transactivate either the epidermal growth factor receptor (EGFR) or the platelet-derived growth factor receptor β (PDGFRβ). Upon GPCR stimulation, these transactivated RTKs exhibit increased tyrosine phosphorylation, as seen similarly following growth factor-induced activation. The transactivation of EGFR by the β_2 _adrenergic receptor is also characterized by increased dimerization of EGFR [8]. In many cases, the transactivation of EGFR is mediated in either a paracrine or autocrine fashion by the metalloproteinase-dependent release of heparin-binding (HB)-EGF. Hence, the mechanism of EGFR activation by GPCRs is similar to that by its own ligand.

Previous work from our laboratory and our collaborators has demonstrated the DRD4-mediated transactivation of PDGFRβ in hippocampal neurons [13] as well as in DRD4-expressing CHO-K1 cells [5]. Despite speculation of a similar mechanism to EGFR transactivation, the mechanism of PDGFRβ transactivation is not clear. The present study aims to investigate the mechanism by which the PDGFRβ is transactivated via DRD4 by examining the roles of a paracrine or autocrine mediator, PDGFRβ cross-phosphorylation and PDGFRβ dimerization in this process.

### Experimental Procedures

#### Reagents and antibodies

Recombinant human PDGF-BB was purchased from R&D Systems (Minneapolis, MN, USA). Dopamine, wortmannin and tyrphostin A9 were obtained from Sigma-RBI (St. Louis, MO, USA). AG1295 and GM6001 were purchased from Calbiochem (San Diego, CA, USA). CRM197 was purchased from List Biochemical Laboratories (Campbell, CA, USA). Antibodies raised against β-tubulin, phospho-Shc and the carboxy terminal region of human PDGFRβ from residues 958 to 1106 were obtained from Santa Cruz Biotechnology (Santa Cruz, CA, USA). Antibodies raised against the extracellular domain of human PDGFRβ were obtained in a biotinylated form from R&D Systems (Minneapolis, MN, USA). Antibodies specific to different phosphorylation sites on PDGFRβ were obtained from two different sources. Anti-phospho-PDGFRβ-Tyr716 was from Upstate Biotechnology (Charlottesville, VA, USA), and phosphospecific PDGFRβ antibodies directed against Tyr740, 751, 857, and 1021 were purchased from Santa Cruz Biotechnology (Santa Cruz, CA, USA). General phosphotyrosine antibodies in an unconjugated form (4G10) and in a horseradish peroxidase-conjugated form (PY20) were purchased from Upstate Biotechnology (Charlottesville, VA, USA) and BD Transduction Laboratories (Franklin Lakes, NJ, USA), respectively. Antibodies to ERK1/2 and phospho-ERK1/2 (Thr202/Tyr204) (E10) were obtained from Cell Signaling Technology (Beverly, MA, USA). Anti-FLAG antibody was purchased from Sigma (St. Louis, MO, USA). Peroxidase-conjugated antibodies to mouse and rabbit IgG were purchased from Sigma (St. Louis, MO, USA) and Cell Signaling Technology (Beverly, MA, USA), respectively. Lipofectamine, G418, zeocin, fetal bovine serum, and horse serum were purchased from Invitrogen Life Technologies (Burlington, ON, Canada). Media used in cell cultures were obtained from either Invitrogen Life Technologies (Burlington, ON, Canada) or Sigma (St. Louis, MO, USA).

#### Plasmids

Expression vectors for epitope-tagged DRD4 and PDGFRβ have been described by us previously [5]. The plasmid encoding the FLAG-tagged human PDGFRβ was a gift from Dr. N. J. Freedman (Duke University, NC, USA) [14]. All plasmids were subcloned into either pcDNA3 or pcDNA3.1 vectors (Invitrogen) containing antibiotic resistance genes for selection with either G418 or zeocin, respectively. A carboxyl-terminal truncated human PDGFRβ (C-truncPDGFRβ) was constructed, as reported by Ueno *et al. *[15] for the mouse PDGFRβ, by truncating the wild-type human receptor after amino acid residue 615 [16]. The insert coding for this truncated receptor was cloned into the *Eco*RI and *Xba*I restriction endonuclease sites in pcDNA3.1-Myc-His-A vector (Invitrogen) for C-terminal tagging of the protein and expression in mammalian cells. To construct the GST-Ig4β, the fourth immunoglobulin domain of the mouse PDGFRβ was taken as the region from amino acid residues 314 to 413 [16], based on an alignment with the region reported for the human receptor [17], and cloned into the *Bam*HI and *Eco*RI restriction endonuclease sites in pGEX-3X (Amersham, Little Chalfont, Buckinghamshire, UK). The sequences of the inserts in all the plasmids used in the present study were confirmed by automatic sequencing (CEQ 2000XL, Beckman Coulter).

#### Cell cultures and transfection

CHO-K1 cells were maintained in α-MEM supplemented with 2.5% fetal bovine serum and 2.5% horse serum [5]. CHO-K1 cells stably expressing HA-DRD4 (CHO/DRD4) or FLAG-PDGFRβ (CHO/PR) alone were maintained in the serum-containing medium supplemented with 500 μg/mL G418, and CHO-K1 cells stably expressing both the HA-DRD4 and FLAG-PDGFRβ (CHO/DRD4-PR) were maintained in serum-containing medium supplemented with both 500 μg/mL G418 and 200 μg/mL zeocin.

For assays on cell lines not subject to further transfection, the cells were seeded and grown to 70-90% confluency prior to serum-deprivation. Transfection was performed on 100 mm plates with a mixture containing 36 μL lipofectamine and 8 μg DNA. The mixture was prepared in 0.6 mL OPTI-MEM (Invitrogen) and incubated for 45 minutes at room temperature before another 6.4 mL of OPTI-MEM was added to yield the final transfection mixture. One day prior to transfection, cells were seeded at a density of 2 × 10^6 ^cells per 100 mm. Transfection was allowed to take place for 5 h, after which time an equal volume of 2 × serum-containing α-MEM was added. On the following day, cells intended for assays were replated at 60-80% confluency or at 0.4-1% for establishing stable cell lines. To establish cell lines expressing the desired constructs stably, the transfected cells were allowed to form isolated colonies. Positive colonies were selected by their resistance to G418 or zeocin. The expression of the desired proteins in the isolated colonies was determined by western blotting.

#### siRNA

The two double-stranded RNA constructs containing the PDGFRβ-specific sequences used were PDGFRβ silencer 1: (sense) 5'-GCAUCUUCAACAGCCUCUAtt-3' and (antisense) 5'-CAGAGGCUGUUGAAGAUGCtt-3' and PDGFRβ silencer 2: (sense) 5'-GUGGACUCCGAUACUUACUtt-3' and (antisense) 5'-AGUAAGUAUCGGAGUCCACtt-3'. These oligonucleotides, as well as the scrambled siRNA (cat. # 4611), were purchased from Ambion (Austin, TX). To suppress PDGFRβ expression, CHO/DRD4 cells were transfected with 30 or 100 nM dsRNA using siPORT transfection reagent (Ambion, Austin, TX) according to the manufacturer's instructions. After 72 h, the cells were used for pharmacological manipulation and harvested for western analysis.

#### GST-Ig4 fusion protein blocking experiments

GST and GST-Ig4β fusion proteins were expressed in BL21 *E. coli*. Purification from bacterial lysates was carried out according to the manufacturer's protocol (Amersham). Elution from glutathione-Sepharose beads (Amersham) was performed as described by Omura *et al. *[18]. For cell assays, the purified proteins were applied at 4 μg/mL and pre-incubated for 20 minutes at 37°C prior to agonist stimulation.

#### Cell assays

CHO-K1 cells were serum-deprived for 24 h prior to assays with either PDGF-BB (10 ng/mL) or dopamine (1 μM) for 5 minutes. In experiments using kinase inhibitors, the cells were pre-incubated with the inhibitor or the appropriate vehicle control solution for 1 h before stimulation with the agonists. Following incubation, cells were chilled immediately on ice and washed two times with ice-cold PBS (137 mM NaCl, 2.7 mM KCl, 5.4 mM Na_2_HPO_4_, 1.8 mM KH_2_PO_4_, pH 7.4). Cells were scraped on ice in RIPA buffer supplemented with protease and phosphatase inhibitors (50 mM Tris-HCl, pH 7.5, 150 mM NaCl, 1 mM EDTA, 1 mM EGTA, 1% NP-40, 0.5% deoxycholate, 0.1% SDS, 5 μg/mL aprotinin, 2 μg/mL leupeptin, 1 μg/mL pepstatin A, 1 mM PMSF, 1 mM Na_3_VO_4_, 2.5 mM pyrophosphate·Na, 1 mM β-glycerophosphate). Sodium orthovanadate was activated using the method described by Gordon *et al. *[19]. Lysis was performed at 4°C for 1 h with continuous shaking. An equal amount of protein was taken from each sample for further analysis by immunoprecipitation and/or western blotting.

#### Immunoprecipitation and immunoblotting

Immunoprecipitation was performed by mixing 1-2 μg antibody with RIPA cell lysates. The mixture was shaken for a minimum of 1 h to overnight at 4°C. After addition of 25 μL Protein A/G Plus beads (Santa Cruz), the incubation was allowed to continue for a total of 17-19 h at 4°C. Immunoprecipitates were collected by centrifugation after washing three times with the NP-40 buffer (50 mM HEPES, 250 mM NaCl, 0.5% NP-40, 10% glycerol, 2 mM EGTA, 5 μg/mL aprotinin, 2 μg/mL leupeptin, 1 μg/mL pepstatin A, 1 mM PMSF, 1 mM Na_3_VO_4_, 2.5 mM pyrophosphate·Na, 1 mM β-glycerophosphate).

Immunoblotting with phosphotyrosine antibody was performed with a procedure adapted from Klapper *et al. *[20] to reduce background staining. Briefly, the blocking of non-specific binding on PVDF membranes and the dilution of antibodies was performed in blocking buffer A (10 mM Tris-HCl, 154 mM NaCl, 2.5 mM MgCl_2_, 3% BSA, pH 7.4). Following incubation with antibodies, the membranes were washed twice with the buffer B (10 mM Tris-HCl, 0.9% NaCl, 0.05% MgCl_2_, pH 7.4), once with blocking buffer B supplemented with 0.3% Tween 20, and twice more with the blocking buffer B. Each wash was carried out for 5 minutes on a rocking platform. Blocking buffer C (10 mM Tris-HCl, 150 mM NaCl, 3% BSA, 0.1% Tween 20, pH 7.4) was used in all associated procedures for immunoblotting with biotinylated antibodies, and blotto (10 mM Tris-HCl, 150 mM NaCl, 5% non-fat dry milk, 0.1% Tween 20, pH 7.4) was used for immunoblotting with other antibodies. Working dilutions of antibodies were prepared as recommended by manufacturers.

#### Data Analysis

Densitometry was performed on Storm 860 phosphorimager (Amersham) with ECL Plus chemiluminescent reagent (Amersham). Quantitation was done using ImageQuant 5.0 software (Molecular Dynamics). Curve fitting was performed in GraphPad Prism 3.0 (San Diego, CA, USA). Details pertaining to specific experiments are provided in the legends to the figures.

## Results

Growth factor-activated PDGFRβ results in receptor cross-phosphorylation of tyrosine residues. We have previously shown that, in CHO-K1 cells stably expressing DRD4 (CHO/DRD4), dopamine stimulates the phosphorylation of ERK1/2 in a manner sensitive to the inhibition of the PDGFRβ kinase [5]. Therefore, we initiated our study by examining whether dopamine and the PDGFRβ ligand, PDGF-BB, activate the PDGFRβ in a similar manner. The commercially available antibodies do not reliably detect the phosphorylation of the endogenously expressed hamster PDGFRβ in the CHO/DRD4 cells; therefore, to facilitate the detection of PDGFRβ phosphorylation at the different tyrosine phosphorylation sites, the human FLAG-tagged PDGFRβ was stably transfected into the CHO/DRD4 cells to create CHO/DRD4-PR cells [14]. In CHO/DRD4-PR cells, the DRD4-mediated PDGFRβ and ERK1/2 phosphorylation was inhibited by pre-treatment with the PDGFRβ kinase inhibitors, tyrphostin A9, AG1295, and AG1296 in a similar manner as in the CHO/DRD4 cells (data not shown).

**Figure 1:**
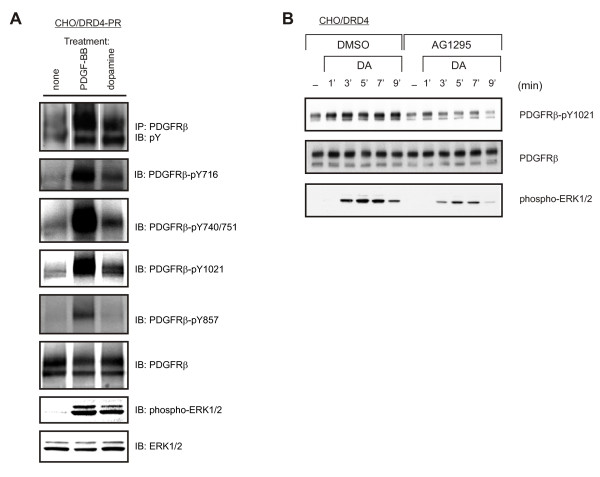
**Phosphorylation of PDGFRβ following DRD4 versus PDGF-BB stimulation**. (A) Differential pattern of phosphorylation of the PDGFRβ elicited by PDGF-BB and DRD4 in CHO-K1 cells overexpressing DRD4 and FLAG-PDGFRβ (CHO/DRD4-PR). (B) Time course of dopamine-stimulated phosphorylation of endogenous PDGFRβ and ERK1/2 in CHO/DRD4 in the presence or absence of AG1295 (10 μM). AG1295 was pre-incubated with the cells for 1 h before agonist stimulation. (A-B) CHO/DRD4 or CHO/DRD4-PR was stimulated for 5 min, or for the time indicated, with 10 ng/mL PDGF-BB or 1 μM dopamine. IP: immunoprecipitation; IB: immunoblot.

Using the CHO/DRD4-PR cells, we compared the pattern of PDGFRβ phosphorylation after stimulation with either 1 μM dopamine or 10 ng/mL PDGF-BB. As shown in Figure 1A, the level of total tyrosine phosphorylation of PDGFRβ was less following dopamine treatment compared to PDGF-BB stimulation. Consistently, using site-specific phospho-antibodies, several SH2 domain-binding sites [Grb2 (Tyr716), PI3-kinase (Tyr740/751) and PLC-γ (Tyr1021)] of the PDGFRβ also showed stronger phosphorylation in response to PDGF-BB compared to dopamine. Interestingly, Tyr857, the major site of tyrosine phosphorylation in PDGF-stimulated cells [21], was phosphorylated only by PDGF-BB, but not dopamine (Figure 1A). The absence of phosphorylation of this site may explain the overall lower tyrosine phosphorylation of PDGFRβ caused by DRD4 stimulation.

Since the receptor kinase activity is not enhanced through Tyr857 phosphorylation, the dopamine-induced PDGFRβ phosphorylation may be a result of the basal kinase activity of the PDGFRβ. The phospho-specific antibody against Tyr1021 of the PDGFRβ does recognize the endogenously expressed hamster receptor; therefore, we used this antibody to measure PDGFRβ phosphorylation in CHO/DRD4 cells. As shown in Figure 1B, the dopamine-induced tyrosine phosphorylation of PDGFRβ (Tyr1021) and the phosphorylation of ERK1/2 were reduced by the PDGFRβ kinase inhibitor AG1295.

**Figure 2:**
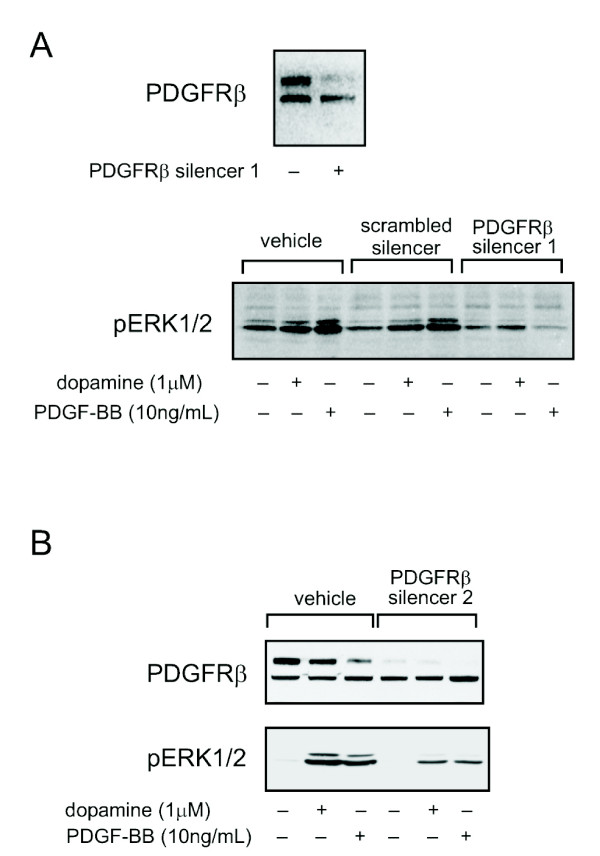
**PDGFRβ siRNAs inhibit dopamine-mediated ERK1/2 activation**. (A) 100 nM of double-stranded PDGFRβ interference RNA (PDGFRβ silencer 1) or (B) 30 nM of double-stranded PDGFRβ RNA (PDGFRβ silencer 2) was transfected into the CHO/DRD4 cells. Cells were harvested at 72 h post transfection and PDGFRβ expression was determined. For phospho-ERK1/2 expression (Thr202/Tyr204), cells were serum-starved overnight (beginning at 48 h post-transfection) and subsequently treated with dopamine (1 βM) or PDGF-BB (10 ng/ml).

To provide direct evidence for a role of the PDGFRβ in dopamine-stimulated ERK1/2 activation, we used siRNA to suppress endogenous PDGFRβ expression in the CHO/DRD4 cells. In these cells, the PDGFRβ exists as two isoforms that can be detected by western blotting: a maturely glycosylated receptor (180 kDa) and a 140 kDa, immaturely glycosylated, isoform [22,23]. In our siRNA experiments, two separate dsRNA constructs were used that showed a similar efficacy in reducing the levels of maturely glycosylated PDGFRβ (Figure 2A, B). We observed that the 140 kDa immaturely glycosylated form of PDGFRβ was not effectively suppressed by either siRNA approach. This band was still present in our western blots when different PDGFRβ antibodies were used; additionally, a similar band was seen in CHO/DRD4 cells transfected with FLAG epitope-tagged PDGFRβ (not shown), suggesting that this band represents a genuine isoform of PDGFRβ. Upon siRNA-mediated PDGFRβ suppression, both dopamine (1 μM) and PDGF-BB (10 ng/ml) treatment showed approximately 50% reduction in ERK1/2 phosphorylation compared to controls. A scrambled dsRNA did not cause a decrease in PDGFRβ expression (data not shown), nor did it suppress ERK1/2 phosphorylation (Figure 2A). These results demonstrate that PDGFRβ may be involved in DRD4-mediated ERK1/2 activation.

**Figure 3:**
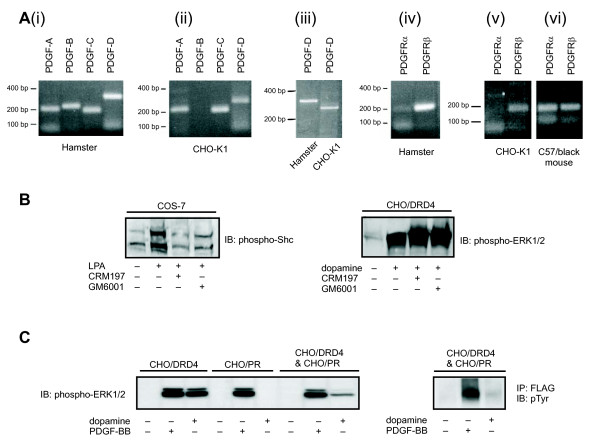
**Absence of a paracrine mediator in DRD4-mediated PDGFRβ transactivation**. (A) mRNA expression of known PDGF ligands and the α and β subtypes of PDGFR. RT-PCR was performed on total RNA extracted from CHO-K1 cells or as controls, on total RNA taken from the cardiac tissues of hamster or C57/black mouse. The primers were designed with the primer3 software to target sequences that are conserved between mouse and human and to yield PCR products of sizes between 200 to 300 bp. Expression of PDGF-A, PDGF-C and PDGFRβ were detected in CHO-K1 cells, with PDGF-D being a non-specific band. (B) CHO/DRD4 or COS-7 cells were treated with the diphtheria toxin mutant CRM197 (10 μg/mL) or the metalloproteinase inhibitor GM6001 (5 μM) for 30 min. The cells were subsequently stimulated with 1 μM dopamine (CHO/DRD4) or 10 μM LPA (COS-7), and lysates were taken for western blotting with phospho-ERK1/2 (CHO/DRD4) or phospho-Shc (COS-7). The inhibitors had no effect on dopamine-stimulated ERK1/2 phosphorylation. (C) CHO/DRD4 and CHO-K1 cells stably expressing FLAG-PDGFRβ (CHO/PR) were cultured separately or together at 90% confluency in a ratio of 1:1. The cells were stimulated with 10 ng/mL PDGF-BB or 1 μM dopamine as indicated. Lysates were collected to probe with phospho-ERK1/2 antibody (*left blot*) or taken for immunoprecipitation with anti-FLAG antibody and immunoblotted with phosphotyrosine antibody (pTyr) (*right blot*). IP: immunoprecipitation; IB: immunoblot.

We then tried to examine the existence of PDGFRβ-specific ligand PDGF-B in the CHO-K1 cells. Previous reports have shown that HB-EGF acts as a paracrine factor in the GPCR-induced transactivation of EGFR [24]. In addition, the PDGF-C and PDGF-D isoforms are secreted as latent pro-peptides and can be cleaved to their active states by extracellular proteases. This has led to the speculation that either PDGF or a PDGF-like ligand may mediate GPCR-induced PDGFRβ transactivation via a similar metalloproteinase-dependent process. Thus, to explore any involvement of a paracrine factor in DRD4-mediated PDGFRβ transactivation, we examined the endogenous expression of PDGFs and PDGFRs in CHO-K1 cells using RT-PCR. As a positive control, the primers were tested in hamster and mouse cardiac tissues and were found to produce PCR products of the expected size (Figure 3Ai, iv, vi). Using the same primers, CHO-K1 cells were found to express PDGF-A and PDGF-C; however, no PDGF-B mRNA was detected in these cells (Figure 3Aii). The PCR products obtained for PDGF-D, using two independent primer sets, did not correspond to that obtained with the control hamster tissues (Figure 3Aiii, and data not shown), suggesting that the band from the CHO-K1 cells was due to non-specific amplification. Sequence analysis confirmed that the observed PDGF-D band was due to non-specific amplification of the presenilin gene. Therefore, CHO-K1 cells express only PDGF-A and PDGF-C, and not PDGF-B or PDGF-D. PDGF-A and PDGF-C are PDGFRα-specific ligands and have been shown to bind PDGFRβ only when PDGFRα is also present [1,25]. Using RT-PCR, it was found that CHO-K1 cells do not express PDGFRα, but do express PDGFRβ (Figure 3Av). These results suggest that in CHO-K1 cells, the transactivation of PDGFRβ by DRD4 stimulation is unlikely to be produced via a paracrine mechanism by known PDGFRβ ligands.

The metalloproteinase inhibitor GM6001 is known to block LPA-mediated Shc phosphorylation in COS-7 cells [12]. Thus, we tested the potential role of metalloproteinases in DRD4-mediated ERK1/2 phosphorylation. As shown in Figure 3A, both GM6001 and the mutant of diphtheria toxin CRM197, which binds HB-EGF and inhibits ectodomain shedding [12], reduced the LPA-induced Shc phosphorylation in COS-7 cells. However, they were ineffective in blocking DRD4-mediated ERK1/2 phosphorylation in CHO/DRD4 cells (Figure 3B). These results suggest there is little or no role of metalloproteinases in DRD4-mediated ERK1/2 phosphorylation.

Although the RT-PCR experiments showed the absence of PDGFRβ-specific ligands, we sought to exclude the possible activation of PDGFRβ by a yet undiscovered ligand. To identify any potential PDGFRβ ligand released upon dopamine stimulation, a co-culture strategy [12,26] in which two different populations of cells were cultured together was employed. We created one population of CHO-K1 cells (CHO/PR), which stably expressed FLAG-tagged human PDGFRβ, but not DRD4. The CHO/DRD4 cell line described above was used as our second population of cells. As expected, when cultured alone, the CHO/PR cells showed ERK1/2 activation in response to PDGF-BB, but not to dopamine, while the CHO/DRD4 cells responded to both dopamine and PDGF-BB (Figure 3C, *left hand side*) when cultured alone. Interestingly, when the two cell populations were co-cultured at a high density in a 1:1 ratio, dopamine-induced ERK1/2 phosphorylation was approximately half as that observed with the CHO/DRD4 monoculture, unlike the PDGF-BB-mediated ERK1/2 response, which was similar in the monocultures and co-culture. We speculated that the reduced dopamine-mediated ERK1/2 phosphorylation in the co-culture is due to the decrease of paracrine mediator, as only half of the cells in this culture express DRD4. Furthermore, immunoprecipitation of the FLAG-tagged PDGFRβ in the CHO/PR cells from the co-culture, followed by immunoblotting of phospho-tyrosine residues revealed an enhanced phosphorylation in response to PDGF-BB, but not dopamine (Figure 3C, *right hand side*). This further suggests that a paracrine mediator of PDGFRβ activation is not released by the CHO/DRD4 cells within the co-culture, in response to dopamine. Taken together, our results from the co-culture and metalloproteinase inhibitor studies suggest that the mechanism of DRD4-mediated transactivation of PDGFRβ does not involve a paracrine factor or it involves a paracrine factor that does not induce transactivation.

**Figure 4:**
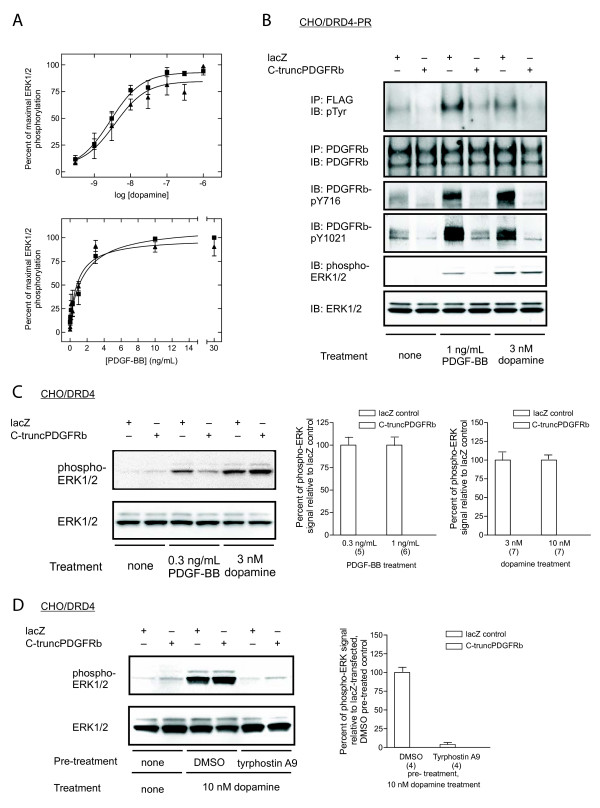
**Cross-tyrosine phosphorylation of PDGFRβ is not required for DRD4-mediated ERK1/2 activation**. (A) Dose-response of DRD4- and PDGF-BB-mediated ERK1/2 phosphorylation. CHO/DRD4 (black square) or CHO/DRD4-PR (black triangle) cells were treated with different concentrations of dopamine or PDGF-BB for 5 min. (B) Effect of C-truncPDGFRβ on the phosphorylation of full-length PDGFRβ and ERK1/2 in CHO/DRD4-PR cells mediated by PDGF-BB and DRD4. CHO/DRD4-PR cells were transfected with C-truncPDGFRβ, or with lacZ as a control. IP: immunoprecipitation; IB: immunoblot. (C) DRD4-mediated phosphorylation of ERK1/2 in CHO/DRD4 was not blocked by C-truncPDGFRβ. (D) Requirement for PDGFRβ in the DRD4-mediated phosphorylation of ERK1/2 in C-truncPDGFRβ-transfected cells. *Upper *blot shows the phosphorylation of ERK1/2 in lacZ-transfected and C-truncPDGFRβ-transfected CHO/DRD4 cells that have been pre-treated with DMSO or 1 μM tyrphostin A9 prior to stimulation with dopamine. (B-D) The blots were stripped and re-probed with antibodies for PDGFRβ or ERK1/2 to verify that the total protein did not vary between lanes. In (C) and (D), the bar graphs show densitometric measurements of the phosphorylation signal from the respective experiments. The results are expressed as percentages relative to lacZ control and indicated as mean ± SEM. The number of experiments is indicated in parentheses.

The observations that PDGF is not involved in DRD4-mediated PDGFRβ transactivation led us to speculate that the DRD4-ERK1/2 pathway is mechanistically different from the growth factor-activated ERK1/2 pathway, although both pathways utilize PDGFRβ. First, we explored the role of PDGFRβ cross-phosphorylation in DRD4- or PDGF-BB-induced ERK1/2 and PDGFRβ phosphorylation. A mouse PDGFRβ mutant with a deletion in the intracellular domain has previously been shown to inhibit PDGF-mediated signaling due to its ability to heterodimerize with the full-length receptor, thereby blocking the formation of functional PDGFRβ dimers and consequently receptor cross-phosphorylation [15]. A similar deletion mutant (see Methods) was created for the human PDGFRβ (C-truncPDGFRβ) and transfected into CHO/DRD4-PR. The cells were lysed and the phosphorylation of ERK1/2 was examined by western blotting with phospho-ERK1/2 antibody (E10). The degree of phosphorylation was quantified using ImageQuant and expressed as percentage of maximal response. The *EC*_50 _values were determined by fitting to a sigmoidal dose-response equation in GraphPad Prism. The log *EC*_50 _values for dopamine were: CHO/DRD4, -8.56 ± 0.11 (*n *= 4); CHO/DRD4-PR, -8.44 ± 0.23 (*n *= 3). The log *EC*_50 _values for PDGF-BB were: CHO/DRD4, -10.13 ± 0.16 (*n *= 4); CHO/DRD4-PR, -10.41 ± 0.15 (*n *= 3). Assays were carried out at or near EC_50 _concentrations of dopamine and PDGF-BB so that a false negative inhibition due to over-stimulation of the signaling pathway could be avoided (Figure 4A).

The effect of C-truncPDGFRβ was examined by western blotting with general phospho-tyrosine or site-specific phospho-antibodies as indicated. To prevent the effect from being masked by signal amplification, the cells were stimulated with submaximal concentrations of either PDGF-BB or dopamine. Immunoprecipitation with anti-FLAG antibody was performed prior to blotting with general phospho-tyrosine antibodies. Relative to the lacZ control plasmid, transfection of C-truncPDGFRβ reduced basal PDGFRβ general tyrosine phosphorylation, as well as receptor phosphorylation in response to both PDGF-BB and dopamine (Figure 4B). A corresponding reduction was also observed at two of the SH2 domain-binding sites. The phosphorylation of the Grb2 site (Tyr716) and the major PLC-γ binding site (Tyr1021) in unstimulated CHO/DRD4-PR and those stimulated by either PDGF-BB or DRD4 activation was reduced by the C-truncPDGFRβ to near basal levels (Figure 4B). Similar to its effect on PDGF-stimulated PDGFRβ tyrosine phosphorylation, the C-truncPDGFRβ also blocked ERK1/2 phosphorylation in response to PDGF-BB. In contrast, the DRD4-mediated ERK1/2 phosphorylation was unaffected by expression of C-truncPDGFRβ. These results suggest that unlike PDGF-mediated signaling, DRD4-induced ERK1/2 phosphorylation is not contingent on PDGFRβ cross-phosphorylation.

We further confirmed that the differential effect of the C-truncPDGFRβ on PDGF- and DRD4-mediated signaling was not due to signal amplification caused by our overexpression of recombinant PDGFRβ by examining ERK1/2 phosphorylation in CHO/DRD4 cells, which express the PDGFRβ endogenously. Consistent with our observations in CHO/DRD4-PR cells, the C-truncPDGFRβ blocked PDGF-BB-mediated ERK1/2 phosphorylation in CHO/DRD4 cells (Figure 4C). In contrast, C-truncPDGFRβ did not inhibit the ERK1/2 phosphorylation that was stimulated by submaximal concentrations of dopamine. To ascertain that the lack of effect of C-truncPDGFRβ on DRD4-mediated ERK1/2 phosphorylation is not due to overexpression of PDGFRβ, the experiment was also performed in CHO/DRD4. The *p *values for the 0.3 ng/mL and 1 ng/mL PDGF-BB-treated groups are 0.02 and 0.01, respectively, according to the paired t-test. Moreover, in the presence of C-truncPDGFRβ, the DRD4-mediated ERK1/2 phosphorylation remained sensitive to PDGFR kinase inhibition, showing that basal kinase activity of PDGFRβ is still required for DRD4-mediated PDGFRβ transactivation (Figure 4D).

**Figure 5:**
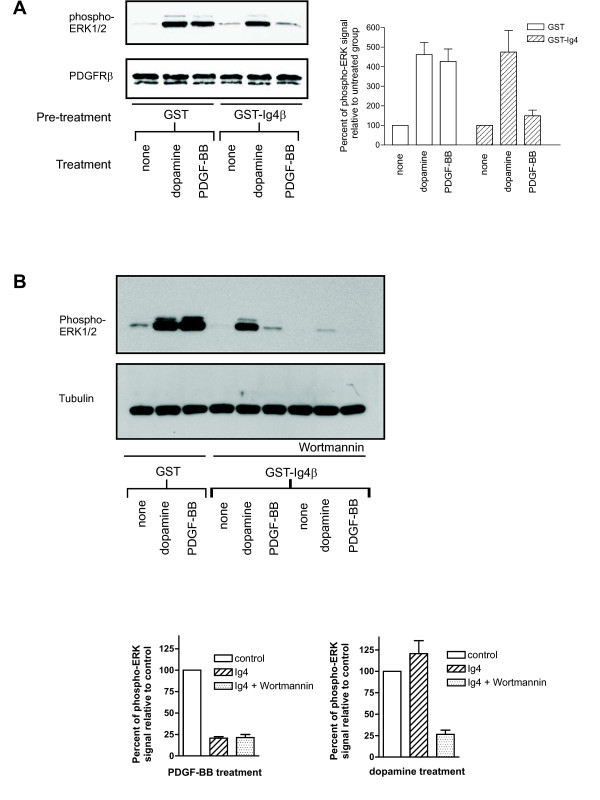
**Blocking PDGFRβ dimerization inhibits ERK1/2 activation in PDGF-BB-treated, but not in dopamine-stimulated CHO-K1 cells**. (A) CHO/DRD4-PR cells were pretreated with 4 μg/mL of either GST or GST-PDGFRβ immunoglobulin domain 4 (GST-Ig4β) fusion proteins for 20 min at 37°C to prevent PDGFRβ dimerization and were then stimulated with 1 μM dopamine or 10 ng/mL PDGF-BB for 5 min. Lysates were taken for western blotting with phospho-ERK1/2 antibody (*upper blot*). The same blot was stripped and reprobed for total PDGFRβ to demonstrate equal loading of all lanes (*lower blot*). (B) Pre-treatment with 100 nM wortmannin for one hour abolished the dopamine-mediated ERK1/2 signal following PDGFRβ dimerization block with GST-Ig4β. The same blot was stripped and reprobed for β-tubulin to demonstrate equal loading of all lanes (*lower blot)*. The bar graphs show the densitometric measurement of the relative signals from phospho-ERK1/2 over two to four experiments, and the quantities were given as mean ± SEM.

The lack of the need for PDGFRβ cross-phosphorylation in DRD4-mediated ERK1/2 activation suggests that PDGFRβ dimerization is also not required. We investigated the need for receptor dimerization, by utilizing a glutathione *S*-transferase fusion protein to inhibit the formation of PDGFRβ dimers. Previous reports have shown that a glutathione *S*-transferase-PDGFRα-Ig4 domain fusion protein (GST-Ig4α) perturbs PDGFRα dimerization [18]. The extracellular Ig4 domain of PDGFRβ is known to provide the interface for subunit-subunit interaction without playing a role in ligand binding [17,18,27,28]. Therefore, to specifically block PDGFRβ dimerization, a glutathione *S*-transferase-PDGFRβ-Ig4 (GST-Ig4β) fusion protein was constructed. Pre-incubation of CHO/DRD4-PR cells with GST alone had little effect on the ability of dopamine or PDGF-BB to elicit an ERK1/2 response (Figure 5). However, incubation with GST-Ig4β prevented PDGF-BB-stimulated ERK1/2 phosphorylation. Conversely, blocking PDGFRβ dimerization with GST-Ig4β did not affect DRD4-mediated ERK1/2 phosphorylation. These results suggest that DRD4 can activate ERK1/2 by utilizing the PDGFRβ in a way that does not require receptor dimerization.

Previous data from our laboratory [5] suggested the involvement of PI3-kinase in the DRD4-mediated activation of PDGFRβ. In order to determine whether PI3-kinase plays a role in the DRD4-stimulated activation of ERK1/2 following the block of PDGFRβ dimerization, CHO/DRD4-PR cells were pre-treated with 100 nM wortmannin for one hour prior to incubation with GST or GST-Ig4β and subsequent treatment with dopamine or PDGF-BB. Wortmannin inhibited the DRD4-mediated ERK1/2 activation observed following PDGFRβ dimerization block with GST-Ig4β, suggesting a role for PI3-kinase in this pathway.

## Discussion

The present study has demonstrated a novel mechanism for PDGFRβ signaling, in which DRD4-mediated transactivation of PDGFRβ and the subsequent activation of ERK1/2 does not involve mechanisms that are characteristic of RTK activation. This new scheme breaks away from the prototypical model, where GPCR-mediated RTK transactivation is ligand-dependent, and so occurs similarly to classical RTK signaling involving receptor dimerization and cross-phosphorylation.

The use of RT-PCR failed to detect any of the known endogenous PDGFRβ ligands within our CHO-K1 cells (Figure 3A). Additionally, inhibition of metalloproteinases failed to suppress DRD4-mediated ERK1/2 activation (Figure 3B), and no evidence of a paracrine mediator was found in DRD4-PDGFRβ transactivation, as demonstrated by our co-culture experiments (Figure 3C). Furthermore, phosphorylation of Tyr857 of the PDGFRβ, a hallmark of ligand-induced activation, was not seen after dopamine treatment (Figure 1A). These lines of evidence argue strongly against the involvement of a paracrine-mediated event in the DRD4-PDGFRβ-ERK1/2 pathway.

Furthermore, dimerization and subsequent cross-phosphorylation of PDGFR are also not required for DRD4-mediated transactivation. DRD4 stimulation led to increased general tyrosine phosphorylation of the PDGFRβ (Figure 1). However, inhibition of the PDGFRβ cross-tyrosine phosphorylation with the C-truncPDGFRβ did not affect dopamine-induced ERK1/2 activation (Figure 4). Similarly, blocking PDGFRβ dimerization with a GST-Ig4β fusion protein did not diminish DRD4-mediated ERK1/2 phosphorylation (Figure 5). Both lines of evidence point to a mechanism that does not require either dimerization or cross-phosphorylation which are hallmarks of RTK activation. Interestingly, wortmannin inhibits the DRD4-mediated ERK1/2 activation observed following PDGFRβ dimerization block with GST-Ig4β (Figure 5B), suggesting a role for PI3-kinase in this pathway.

In the context of transactivation, the PDGFRβ tyrosine phosphorylation that follows DRD4 stimulation appears to be unrelated to the ERK1/2 signaling pathway. It is not clear how the transactivated PDGFRβ mediates signaling in the absence of enhanced tyrosine phosphorylation. The sensitivity of DRD4-mediated ERK1/2 signaling to PDGFRβ kinase inhibitors suggests that a certain level of basal kinase activity is required. An alternative explanation is that through a tyrosine phosphorylation-independent conformational change, the PDGFRβ may act as a scaffold to mediate DRD4-PDGFRβ-ERK1/2 signaling. In fact, growth factor-activated PDGFRβ is known to undergo conformational changes that can be either enhanced by mutations that result in constitutive activity [29] or those that suppress kinase activity [30]. Interestingly, several studies have revealed a mechanistic intricacy of PDGFRβ signaling beyond a simple relationship of dimerization and cross-phosphorylation. There is evidence that PDGF-activated mitogenic responses and receptor tyrosine phosphorylation do not always correlate [31-33].

The present study shows that unlike the prototypical mechanism underlying GPCR-RTK transactivation, DRD4-PDGFRβ-ERK1/2 signaling does not involve a paracrine component, nor does it require PDGFRβ cross-phosphorylation and dimerization. This suggests that PDGFRβ can act as a monomeric scaffold to transmit DRD4-mediated signals, in a tyrosine phosphorylation-independent manner. We have recently demonstrated that DRD4 is able to transactivate immaturely glycosylated PDGFRβ, which is intracellularly localized [34]. These findings preclude the involvement of an extracellular ligand-mediated mechanism of PDGFRβ activation, and would allow DRD4 to remain engaged in the ERK1/2 signaling pathway, despite desensitization of plasma membrane-expressed PDGFRβ. The actual mechanism of how DRD4 stimulation induces PDGFRβ transactivation is still unknown, but we speculate that it involves a diffusable factor, such as PI3-kinase, that would be able to act on a monomeric, intracellularly localized PDGFRβ. The DRD4-PDGFRβ-ERK1/2 pathway is distinct from other known forms of transactivation, and so represents a novel system that already has implications in the regulation of downstream effectors such as the NMDA receptor.

## Competing interests

The authors declare that they have no competing interests.

## Authors' contributions

CSS did the RT-PCR, dose-response curves, and c-truncated PDGF receptor experiments. SMV carried out the GST fusion protein work. RSG performed the siRNA studies. MSH carried out the immunoprecipitation and Western blotting experiments for PDGF receptor phosphorylation. Both SMV and CSS participated in the experimental design and writing of the manuscript. FL contributed to important discussions and manuscript writing. The project was initially under the supervision of the late Dr. Van Tol who passed away in April 2006. All authors read and approved the final manuscript.
